# Geographic Atrophy Progression in Clinical Practice Before and After Pegcetacoplan Treatment

**DOI:** 10.3390/vision9040095

**Published:** 2025-11-18

**Authors:** Jessica A. Cao, Avery W. Zhou, Gail M. Teagle, Liisa M. Baumann, Ryan A. Sahraravand, Calvin W. Wong, Sandro De Zanet, Natasa Jovic, Patrick Steiner, Sagar B. Patel, Samuel A. Minaker, Mathew W. MacCumber, David M. Brown, Hasenin Al-khersan, Charles C. Wykoff

**Affiliations:** 1 Retina Consultants of Texas, Bellaire, TX 77401, USAasahra78@gmail.com (R.A.S.);; 2 Ruiz Department of Ophthalmology and Visual Science, The University of Texas Health Science Center at Houston, Houston, TX 77030, USA; 3 Ikerian AG, 3010 Bern, Switzerland; 4 Department of Ophthalmology, Rush University Medical Center and Illinois Retina Associates, Chicago, IL 60612, USA; samuel.minaker@illinoisretina.com (S.A.M.); mmaccumber@illinoisretina.com (M.W.M.); 5 Blanton Eye Institute, Houston Methodist Hospital, Houston, TX 77030, USA

**Keywords:** geographic atrophy, age-related macular degeneration, pegcetacoplan, artificial intelligence

## Abstract

This retrospective study evaluated changes in ocular characteristics and retinal pigment epithelium (RPE) and photoreceptor ellipsoid zone (EZ) depletion rates before and after intravitreal pegcetacoplan initiation in clinical practice. A total of 168 eyes from 110 patients with GA secondary to age-related macular degeneration (AMD) who received at least 3 pegcetacoplan injections were included. Data was collected from 5 years before to 9 months after pegcetacoplan initiation. RPE and EZ depletion areas were measured using an automated artificial intelligence (AI) algorithm on optical coherence tomography (OCT) images. At baseline, 76 eyes (45.2%) had concurrent neovascular AMD (nAMD), with mean RPE and EZ depletion areas of 3.3 mm^2^ and 4.9 mm^2^, respectively. By pegcetacoplan initiation, these increased to 8.6 mm^2^ and 11.2 mm^2^, respectively, with 151 eyes (89.9%) having concurrent nAMD and 155 eyes (92.3%) having subfoveal GA. Pre-treatment to post-treatment RPE and EZ square root depletion rates decreased from 0.25 mm/year to 0.096 mm/year, and 0.26 mm/year to 0.049 mm/year, respectively. Mean best-recorded visual acuity (BRVA) worsened by 0.05 logMAR annually before and after treatment. These real-world findings align with data from the pegcetacoplan phase 3 trials, showing reduced RPE and EZ depletion rates without changes in rates of BRVA loss. Additional studies are warranted.

## 1. Introduction

Geographic atrophy (GA) and neovascular age-related macular degeneration (AMD) are advanced forms of AMD that together represent leading causes of irreversible vision loss [1]. GA is characterized by the progressive loss of the retinal pigment epithelium (RPE), photoreceptors (PR), and choriocapillaris [2,3,4]. GA currently affects over 5 million people globally, with prevalence estimates expected to rise to nearly 20 million by 2040 [5]. The risk of developing GA increases exponentially with age [5,6,7]. Patients with GA often experience a lower quality of life due to reduced visual acuity (VA), contrast sensitivity, and reading speed [8,9].

GA is typically diagnosed using non-invasive imaging techniques, including fundus autofluorescence (FAF), optical coherence tomography (OCT), and fundus photography (FP) [10]. OCT is particularly beneficial due to its high patient tolerance, reliability, and ability to provide detailed, quantifiable structural data [11,12,13,14]. For these reasons, OCT has become the preferred imaging modality for longitudinal GA studies. In 2018, OCT was proposed as the gold standard for diagnosing GA, and specific criteria for recognizing complete RPE and outer retinal atrophy (cRORA) were proposed [15]. Although clinicians are capable of diagnosing and managing GA using ophthalmoscopy in combination with qualitative OCT analysis, consistent, reproducible, quantitative OCT interpretation often requires either a central reading center and/or assistance from algorithms.

The use of artificial intelligence (AI) to interpret retinal imaging of AMD patients has been investigated in various settings [16,17], such as detecting GA from color fundus photographs [18,19] and assessing AMD severity [20,21]. AI has also demonstrated accuracy in measuring GA lesion size and growth rate with OCT, making it a valuable tool in both research and potentially in clinical practice [22]. RetinAI Discovery, an AI software (Ikerian AG, Bern, Switzerland), has been used to locate the fovea, quantify retinal fluid volumes, segment GA lesions, and measure depletion of specific retinal layers [23,24]. Quantitative automated algorithm interpretation of OCT data in the context of routine clinical practice has the potential to improve disease detection and allow more precise assessment of disease progression and changes in the natural history with intervention [17,25].

Several intrinsic and extrinsic factors have been implicated in GA pathogenesis, including overactivation of the complement cascade [26,27]. Pegcetacoplan (Syfovre, Apellis, Waltham, MA, USA), an inhibitor of C3b and C3 cleavage, was approved by the US Food and Drug Administration (FDA) in February 2023 for the treatment of GA at a dosing interval of once every 25 to 60 days [28,29]. Through two years in the OAKS and DERBY phase 3 clinical trials, pegcetacoplan injections slowed GA lesion growth by 18% to 22% [30]. At enrollment, all 1501 eyes in the phase 2 and 3 FILLY, OAKS, and DERBY trials had GA in the study eye with no history of neovascular AMD (nAMD) in that eye [30,31]. In comparison, GA and nAMD often co-exist in the same eye; approximately a quarter of all new diagnoses of nAMD have concurrent GA [32], and approximately 25% of GA cases without nAMD at baseline are diagnosed with nAMD within 3 years [33]. Therefore, there remains a major unmet need to better understand the effect of pegcetacoplan on GA lesion growth in routine clinical practice, especially among this previously excluded subgroup of eyes with concurrent nAMD.

The current analysis assessed the ocular characteristics and RPE and photoreceptor ellipsoid zone (EZ) depletion rates among a cohort of patients treated with pegcetacoplan in routine clinical practice.

## 2. Materials and Methods

Data was retrospectively collected from electronic health records from a large, urban retina practice (Retina Consultants of Texas, Houston, TX, USA). Inclusion criteria were eyes diagnosed with GA that received at least three pegcetacoplan injections before the start of data collection, with at least five years of clinical data before initiating pegcetacoplan. No other exclusion criteria were used. Institutional review board (Advarra Institutional Review Board, Columbia, MD, USA) approval was obtained (Pro00073225). All analysis procedures adhered to the tenets outlined in the Declaration of Helsinki and the Health Insurance Portability and Accountability Act.

Clinical data collected from medical records (Nextech Systems, LLC, Tampa, FL, USA) included demographics and medical history; ocular characteristics such as best-recorded VA (BRVA), AMD status, and intraocular pressure (IOP); and treatment history and interval. BRVA was categorized by the number of eyes at each time point that progressed to Snellen 20/200 or worse and 20/40 or worse as reported previously [4]. AMD status was categorized as GA, nAMD, or GA with concurrent nAMD. Foveal involvement was assessed using multimodal imaging by three graders.

For each eye, spectral domain-OCT scans (Spectralis, Heidelberg Engineering, Franklin, MA, USA) were captured with 512 A-scans per B-scan and either 60 μm spacing or 124 μm spacing between each B-scan in 97- or 49-line scans, respectively. All patient OCT images were initially captured as 49-line scans, then updated to 97-line scans in 2023. All macular scans were 20° × 20°, covering an analysis area of approximately 36 mm^2^ per scan. OCT images were analyzed using a segmentation model deployed on RetinAI Discovery (Ikerian AG, Bern, Switzerland) and the output included the depletion area of the RPE and depletion area of the ellipsoid zone (EZ) (comprising the EZ, outer photoreceptor segment [OPR], and interdigitation zone [IZ]). The EZ appears as a hyperintense band on OCT due to the dense accumulation of light-scattering photoreceptor mitochondria [34]. Degradation of the EZ (discontinuities, thinning, and decreased reflectivity/brightness) reflects the deterioration of photoreceptors in a quantifiable way [35], and has been approved by the FDA as a primary outcome parameter of GA trials [36]. EZ thickness measurements were made from the inner border of the EZ to the inner border of the RPE, and depletion of the EZ was defined as thickness < 5 µm.

The RetinAI segmentation model was trained using high-quality, manually annotated OCT images to segment key retinal layers—such as the myoid zone ([MZ], external limiting membrane to EZ), EZ + OPR + IZ (PR), and RPE—and biomarker structures like sub-RPE material, drusen, and fluid. Expert graders annotated these images from a longitudinal dataset of patients with GA, intermediate AMD, neovascular AMD, and other retinal diseases. These annotations served as inputs to a Convolutional Neural Network (CNN), enabling the development of an automated segmentation model that accurately measures thickness, volume, and defects [37,38]. This algorithm is listed in the European Database on Medical Devices and has been regulatory approved and Conformité Européenne marked according to the European Union Medical Device Regulation and Medical Devices Directive [39,40]. Among patients with atrophic AMD, when compared with two expert graders, the algorithm achieved mean Dice scores of 0.881 and 0.844, sensitivity of 0.850 and 0.915, and precision of 0.928 and 0.799, respectively [37]. The updated, research use only advanced segmentation algorithm used in this study has shown strong correlations between manually annotated lesion area and AI-identified areas of RPE, PR, and MZ loss within an initial sample of 180 OCT scans [41].

Clinical and imaging data were collected from 17 October 2017 to 1 April 2024, reflecting a timeframe starting five years before pegcetacoplan initiation (defined as baseline) to 9 months after pegcetacoplan initiation. The frequency of data collection increased from annually at baseline to quarterly at year 3.5, resulting in a total of 14 total time points. Visits prior to pegcetacoplan initiation were classified as the pre-dosing phase, and visits following pegcetacoplan initiation were classified as the post-dosing phase. Only patients who met all inclusion criteria and had available clinical data at all 14 time points were included in this report.

Statistical analyses were performed using RStudio software, version 4.3.3 (www.rstudio.com, accessed on 19 March 2024; Boston, MA, USA), and Microsoft Excel, version 16.84 (Microsoft Corporation, Redmond, WA, USA). Snellen BRVA fractions were converted to the logarithm of the minimum angle of resolution (logMAR) for analysis [42]. Square root transformations were performed on lesion area measurements to minimize the effect of baseline lesion size on subsequent growth rate [43]. Student’s paired *t*-test and Welch’s *t*-test were used to determine statistically significant differences between means, with a *p*-value of less than 0.05 considered statistically significant.

## 3. Results

**Figure 1:**
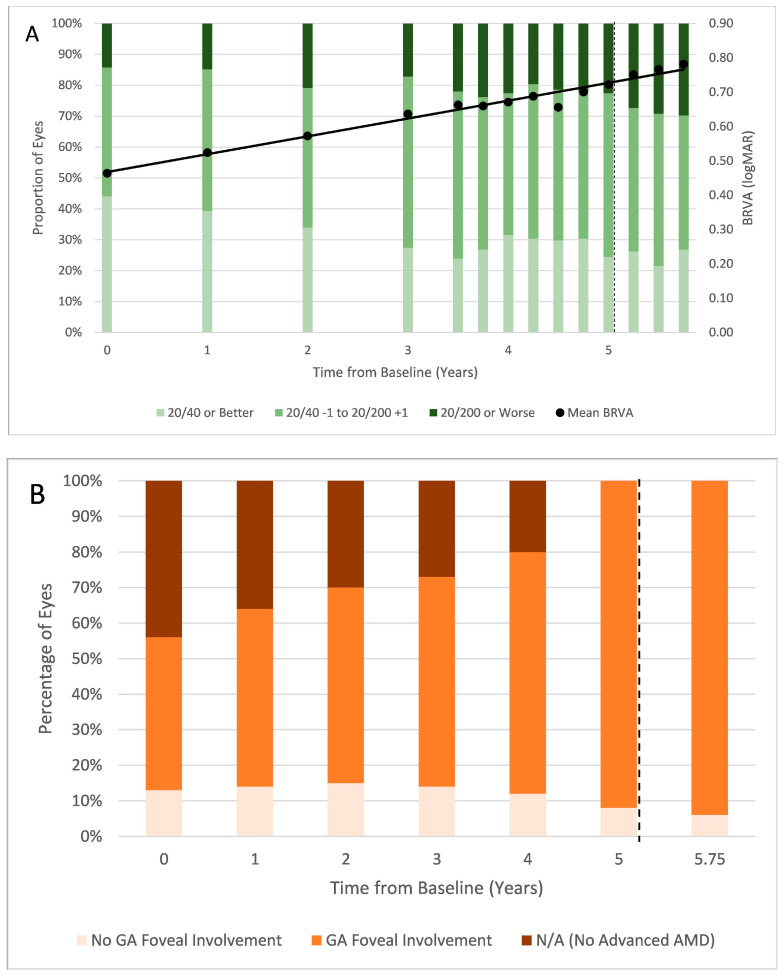

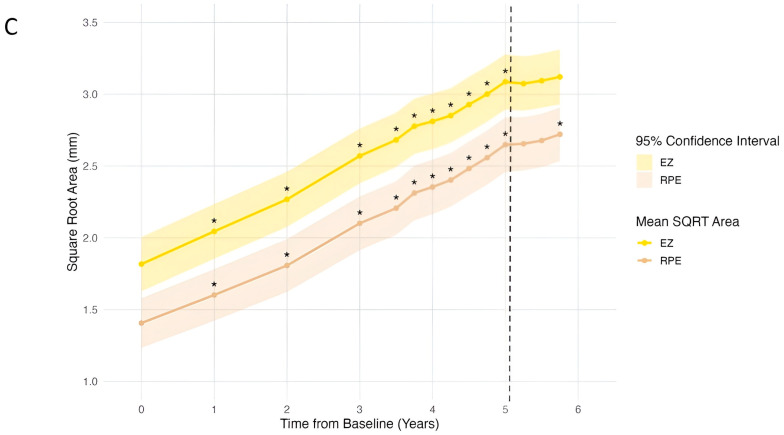
Changes in (**A**) proportion of eyes with various ranges of BRVA and mean BRVA at each time point; solid black line represents the linear best fit of mean BRVA trajectory throughout the clinical course. (**B**) GA foveal involvement. (**C**) square root transformation of RPE and EZ depletion areas from baseline (5 years before pegcetacoplan initiation) through 9 months following pegcetacoplan initiation; * signifies statistical significance of *p* < 0.05 between the indicated point and the previous point on two-tailed *t*-test. In each figure component, the dotted line represents the date of pegcetacoplan initiation. BRVA = best-recorded visual acuity; logMAR = logarithm of the minimum angle of resolution; GA = geographic atrophy; RPE = retinal pigment epithelium; EZ = ellipsoid zone; SQRT = square root.

In total, 168 eyes from 110 patients met the inclusion criteria and had clinical data at all 14 time points (Table 1 and Table 2). Ocular characteristics from baseline through 9 months after pegcetacoplan initiation are shown in Figure 1.

### 3.1. AMD Phenotype

Of all 168 eyes, 76 (45.2%) had both GA and nAMD at baseline (year zero [Y0]), 56 (33.3%) had nAMD only at baseline and were later diagnosed with GA, 18 (10.7%) had GA only at baseline, and 18 (10.7%) had intermediate AMD at baseline and were later diagnosed with GA. At pegcetacoplan initiation (year five [Y5]), 17 eyes (10.1%) had GA without nAMD while 151 eyes (89.9%) had GA with concurrent nAMD. By the end of the study period, 2 of the 17 eyes (11.7%) with only GA at pegcetacoplan initiation had developed concurrent nAMD, resulting in 15 eyes (8.9%) having GA without nAMD and 153 eyes (91.1%) having GA with concurrent nAMD at the end of the study period. Among eyes with intermediate AMD at baseline, 16 of the 18 (88.9%) eyes had GA only at pegcetacoplan initiation, while 2 of the 18 eyes (11.1%) had GA with concurrent nAMD.

### 3.2. BRVA and Foveal Involvement

At baseline, mean (standard deviation [SD]) BRVA was 0.49 (0.47) logMAR (approximately 20/63 Snellen). At the time of pegcetacoplan initiation, mean (SD) BRVA was 0.72 (0.62) logMAR (approximately 20/100 Snellen) and 9 months after pegcetacoplan initiation, mean (SD) BRVA was 0.76 (0.64) logMAR (approximately 20/125 Snellen). Throughout follow-up (Figure 1A), BRVA declined at a mean rate of 0.05 logMAR (approximately 2.5 letters) per year (R^2^ = 0.97), a rate that was consistent during both the pre- and post-pegcetacoplan dosing phases. Over the 5.75-year follow-up period, a total of 14 mean letters were lost and over the different time periods the rates of BRVA loss were comparable among all sub-groups.

At baseline, 73 of the 94 eyes (77.7%) had GA involving the fovea (Figure 1B). At pegcetacoplan initiation, foveal involvement had increased to 155 of 168 eyes (92.3%), and this further increased to 158 of 168 eyes (94.0%) by the end of the study period.

### 3.3. Treatment Characteristics

Following pegcetacoplan initiation, there were a mean (SD) of 6.1 (1.4) pegcetacoplan injections given over 9 months, with a mean interval between injections of 7.4 (0.9) weeks. Five eyes (3.0%) experienced adverse events, including two eyes (11.7% of the 17 eyes without nAMD at pegcetacoplan initiation) that developed nAMD and were treated with anti-vascular endothelial growth factor-A (VEGF) injections. One eye (0.6% of all 168 eyes) developed culture-positive endophthalmitis, which resolved after treatment with intravitreal antibiotics. Additionally, two eyes (1.2% of all 168 eyes) developed intraocular inflammation without vasculitis.

During the pre-dosing phase, and reflecting the studied population with a majority of eyes having concurrent nAMD, 151 eyes (90.0% of all 168 eyes) received anti-VEGF injections. During the post-dosing phase, 2 of these eyes did not receive additional anti-VEGF injections due to nAMD inactivity and 2 eyes initiated anti-VEGF treatment after development of nAMD. Most eyes received anti-VEGF therapy on the same day as pegcetacoplan. Among the eyes treated with anti-VEGF therapy during both the pre-dosing and post-dosing phases, the mean (SD) interval between anti-VEGF injections decreased from 7.6 (2.7) weeks before pegcetacoplan initiation to 6.7 (1.2) weeks afterwards (*p* = 0.01) (Table 3).

### 3.4. Progression of OCT Characteristics

The progression of RPE and EZ area depletion is shown in Figure 1C. At baseline, the mean (SD) depletion area was 3.28 mm^2^ (4.62 mm^2^) for RPE and 4.86 mm^2^ (5.99 mm^2^) for EZ. At pegcetacoplan initiation 5 years later, the mean depletion areas had increased to 8.61 mm^2^ (6.86 mm^2^) for RPE (*p* < 0.001) and 11.19 mm^2^ (7.94 mm^2^) for EZ (*p* < 0.001). The mean rates of depletion in the pre-dosing phase for the RPE and EZ were 1.1 mm^2^/year (R^2^ = 0.98, 95% confidence interval [CI] [0.95–1.2]) and 1.3 mm^2^/year (R^2^ = 0.99, 95% CI [1.2–1.4]), respectively; the square root transformation of RPE and EZ depletion rates were 0.25 mm/year (R^2^ = 0.995, 95% CI [0.24–0.26]) and 0.26 mm/year (R^2^ = 0.998, 95% CI [0.25–0.26]), respectively.

In the nine months prior to initiating pegcetacoplan treatment, the RPE and the EZ experienced a mean depletion of 1.3 mm^2^ and 1.5 mm^2^, respectively. This corresponded to a square root area mean of 0.25 mm for both the RPE and EZ. Following pegcetacoplan initiation, the depletion of the RPE and EZ decreased by a mean of 0.4 mm^2^ and 0.2 mm^2^, respectively, with square root area means of 0.07 mm and 0.03 mm.

Based on this data, there appeared to be a decrease in the rate of depletion for both the RPE and EZ after pegcetacoplan initiation. Considering the entire study period, on an annual basis, the RPE depletion rate decreased from 1.1 mm^2^/year to 0.48 mm^2^/year (R^2^ = 0.73, 95% CI [−0.40–1.4]), while the EZ depletion rate decreased from 1.3 mm^2^/year to 0.29 mm^2^/year (R^2^ = 0.40, 95% CI [−0.77–1.3]). The square root transformation of RPE and EZ depletion rates decreased from 0.25 mm/year to 0.096 mm/year (R^2^ = 0.89, 95% CI [0.00–0.20]) and from 0.26 mm/year to 0.049 mm/year (R^2^ = 0.64, 95% CI [−0.06–0.16]), respectively. Overall, square root RPE depletion and EZ depletion rates decreased by 63% and 81%, respectively, following pegcetacoplan initiation. At the nine-month mark following pegcetacoplan initiation, the mean (SD) depletion areas for the RPE and EZ were 8.96 (6.91) mm^2^ and 11.38 (7.87) mm^2^, respectively.

A sub-analysis of the small subset of eyes without nAMD at pegcetacoplan initiation (sample size [*n*] = 17) revealed similar changes in progression rates. Depletion rates for the RPE and EZ among these eyes decreased by 56% and 77%, respectively, following pegcetacoplan initiation. Square root transformations of the RPE and EZ depletion rates decreased from 0.25 mm/year (R^2^ = 0.99, 95% CI [0.25–0.26]) to 0.16 mm/year (R^2^ = 0.38, 95% CI [−0.46–0.79]), and from 0.25 mm/year (R^2^ = 0.99, 95% CI [0.24–0.26]) to 0.12 mm/year (R^2^ = 0.28, 95% CI [−0.44–0.67]), respectively; the square root transformations of RPE and EZ depletion rates decreased by 36% and 52%, respectively.

## 4. Discussion

This retrospective analysis evaluated changes in ocular characteristics and OCT-based RPE and EZ depletion areas in eyes with GA that were treated with pegcetacoplan in routine clinical practice. While the outcomes from this retrospective analysis were overall consistent with key findings from the phase 3 clinical trials OAKS and DERBY [30], the current population was distinct from the patients enrolled in the pegcetacoplan development program, as the large majority of patients in this real-world analysis had GA with foveal involvement and concurrent nAMD. This subgroup is of particular interest as previous analyses only enrolled patients with GA without nAMD.

In the current work, following pegcetacoplan initiation, the rates of RPE and EZ depletion decreased. Retrospective analyses of clinical trial data have revealed similar trends among patients with GA without concurrent nAMD. For example, an analysis using a different automated deep learning algorithm assessing RPE and photoreceptor layers of 113 of 246 eyes from the FILLY phase 2 trial found decreases in mean change in photoreceptor area loss following pegcetacoplan treatment [22]. Analysis of the phase 2 FILLY trial data also found that eyes treated monthly or every other month with pegcetacoplan experienced a reduction in photoreceptor area loss at 2, 6, and 12 months compared to sham-controlled eyes [31,44]. Additionally, an independent analysis of 192 eyes from the FILLY trial reported that pegcetacoplan-treated eyes had a significantly thicker outer nuclear layer (ONL) compared to the sham-controlled group at 12 months [45].

Direct comparison of data from clinical trials with data from real-world, routine clinical practice is challenging, especially when there are meaningful differences between the populations analyzed. Most notably, while 90% of the current study population had concurrent nAMD, clinical trials of pegcetacoplan excluded patients with concurrent nAMD in the study eye at the time of enrollment. Therefore, especially given how commonly GA and nAMD are present in the same eye [32,33], the current study provides valuable new information for this clinically relevant cohort intentionally not studied in the phase 2 and 3 clinical trials. This study noted a similar response to pegcetacoplan regardless of nAMD status.

Values for RPE and EZ depletion were reported in the current work rather than GA growth rates. Although the term GA currently carries broader recognition, RPE and EZ depletion more accurately represent the data generated in the current work and the region directly impacted on OCT in the context of GA [15]. Post hoc analyses of OAKS and DERBY by Schmidt-Erfurth et al. reported relationships between EZ and RPE loss and GA progression as well as pegcetacoplan response [46], reinforcing their relevance in eyes with GA. In the current analyses, which are limited due to relatively small sample sizes, there appeared to be a reduction in RPE and EZ depletion rates after pegcetacoplan initiation. However, caution should be applied when interpreting these results, as the confidence intervals assessing the change in depletion rates before and after pegcetacoplan within the current work all included zero.

In the context of this possible anatomic benefit demonstrated by reduced RPE and EZ depletion rates following pegcetacoplan initiation, BRVA worsened over the course of follow-up with no detected change in the rate of BRVA decline post-pegcetacoplan initiation, consistent with the pegcetacoplan clinical trial data. Nevertheless, it is possible that slowing GA progression can ultimately lead to preservation of visual function, as suggested within the prospective GALE long-term follow-up study following the phase 3 trials [47]. Within the current real-world dataset, the 9-month duration of follow-up while receiving pegcetacoplan treatment is relatively limited, and longer-term follow-up of larger patient populations may be useful to more completely understand the impact of pegcetacoplan treatment on visual function longitudinally and in order to identify baseline predictors of response.

One bias to consider when interpreting the current data is the likely approach utilized by treating physicians when adopting this novel therapy into clinical practice; at the time of pegcetacoplan initiation, 92.3% of eyes had foveal involvement, and mean VA was approximately 20/100. Therefore, this dataset with a high baseline disease severity does not reflect the full clinical spectrum of pegcetacoplan-treated eyes and may limit the generalizability of results to eyes with smaller areas of GA.

The relationship between GA and visual function is complex; notably, GA progression is often not directly nor proportionally reflected in change in VA [9]. Central VA is often preserved until late in the progression of GA due to foveal sparing [12,48,49]. Consequently, patients with extensive GA can maintain relatively good VA while experiencing significant difficulties with other aspects of visual function such as near tasks, reading, and functioning in low-light settings [50,51]. Assessment of other functional measures, such as contrast sensitivity and microperimetry, have been shown to correlate more closely with GA morphological changes [30,52,53] but were not collected as part of routine clinical care in the current cohort. Since most patients included in this study had both advanced forms of AMD, the mechanisms of visual decline longitudinally were likely multifactorial across the population. For example, Rosenfeld et al. reported that among eyes with nAMD treated with monthly ranibizumab, the primary characteristics associated with visual decline were RPE abnormalities and atrophic changes [54]. Atrophy and fibrosis appear to be the main drivers of vision loss among patients with nAMD [55]. While the follow-up interval in the current study was limited, no appreciable change in VA trajectory was observed following pegcetacoplan initiation.

Among the 17 eyes that did not have concurrent nAMD at pegcetacoplan initiation, 2 eyes developed concurrent nAMD, comparable with the rates observed in OAKS and DERBY [30]. While no cases of retinal vasculitis were observed in this cohort, this study only included patients who had received 3 or more pegcetacoplan injections and cases of occlusive vasculitis seem to occur after the first injection [56,57]. Thus, this report does not provide a comprehensive safety analysis of pegcetacoplan, as the primary focus was on lesion growth rates, and conclusions related to the safety of pegcetacoplan are restricted.

This retrospective study has important limitations, including the short duration of clinical follow-up of 9 months after pegcetacoplan initiation, which limits conclusions on durability and long-term visual outcomes. Additionally, the retrospective design and single-center cohort reduce the external validity of the results. Another key point is that most of the eyes included in the study had concurrent nAMD with foveal involvement at the time pegcetacoplan treatment was initiated. While this is representative of many real-world patients and provides information about a cohort not studied in the clinical trials, it prevents direct comparison with data from the clinical trials, which excluded eyes with concurrent nAMD. There remains a need for additional studies evaluating the safety and efficacy of pegcetacoplan among eyes with GA with concurrent nAMD at the time of pegcetacoplan initiation. There are also limitations related to the imaging analyses utilized, and additional studies evaluating outcomes with the RetinAI algorithm [22] used to measure areas of retinal layer depletion in this study are warranted.

## 5. Conclusions

In summary, reductions in RPE and EZ depletion rates were observed in a population of patients treated with pegcetacoplan in routine clinical practice, a majority of which had foveal involvement and concurrent nAMD. Despite these apparent anatomic benefits, declines in visual function were also noted over the 9-month pegcetacoplan-dosing phase, consistent with the declines observed before pegcetacoplan initiation. Longer-term data following pegcetacoplan initiation and larger datasets may be useful to more completely understand the impact of pegcetacoplan in clinical practice.

## Figures and Tables

**Table 1:** Patient characteristics at baseline, i.e., 5 years before pegcetacoplan initiation.

Characteristic		Distribution
Total number of patients		110
Total number of eyes		168
Age, mean (SD)		77.9 (10.6)
Sex	Male	28 (25.5%)
	Female	82 (74.5%)
Race	White	38 (34.5%)
	Black	0 (0.0%)
	Asian	1 (0.9%)
	Unknown/Other	71 (64.5%)
Ethnicity	Hispanic or Latino	0 (0.0%)
	Not Hispanic or Latino	74 (67.3%)
	Ethnicity not specified	36 (32.7%)
Smoking Status	Never	60 (54.5%)
	Current	6 (5.5%)
	Former	44 (40.0%)
Diabetes	Yes	18 (16.4%)
	No	91 (82.7%)
	Pre-diabetic	1 (0.9%)
Hypertension	Yes	74 (67.3%)
	No	36 (32.7%)

**Table 2:** Ocular characteristics at baseline, i.e., 5 years before pegcetacoplan initiation.

Characteristic		Distribution
BRVA, logMAR	Mean (SD) [Snellen approximate]	0.49 (0.47) [20/63]
	20/40 or better	74 (44.0%)
	20/40 to 20/200	77 (45.8%)
	20/200 or worse	17 (10.1%)
IOP, mean (SD) mmHg		15.3 (3.3)
Phakic status	Phakic	43 (25.6%)
	Pseudophakic	125 (74.4%)
Glaucoma	Yes	18 (10.7%)
	No	150 (89.3%)
GA + nAMD		76 (45.2%)
GA only		18 (10.7%)
nAMD only		56 (33.3%)
Intermediate AMD only		18 (10.7%)

BRVA = best-recorded visual acuity; IOP = intraocular pressure; SD = standard deviation; GA = geographic atrophy; nAMD = neovascular age-related macular degeneration; AMD = age-related macular degeneration.

**Table 3:** Treatment characteristics throughout the duration of follow-up.

Characteristic		Count
Pegcetacoplan injections, mean (SD)		6.1 (1.4)
Pegcetacoplan injection interval, mean (SD) weeks		7.4 (0.9)
Adverse events after pegcetacoplan initiation	nAMD	2 (11.7%)
	Endophthalmitis	1 (0.6%)
	Intraocular inflammation	2 (1.2%)
Anti-VEGF treatment interval 6 months before pegcetacoplan initiation, mean (SD) weeks		7.6 (2.7)
Anti-VEGF treatment interval 9 months after pegcetacoplan initiation, mean (SD) weeks		6.7 (1.2)

nAMD = neovascular age-related macular degeneration; VEGF = vascular endothelial growth factor-A.

## Data Availability

The original contributions presented in this study are included in the article. Further inquiries can be directed to the corresponding author.

## Abbreviations

The following abbreviations are used in this manuscript:

AI	Artificial intelligence
AMD	Age-related macular degeneration
BRVA	Best-recorded visual acuity
CI	Confidence interval
CNN	Convolutional Neural Network
cRORA	Complete retinal pigment epithelium and outer retinal atrophy
EZ	Ellipsoid zone
FA	Fundus autofluorescence
FDA	Food and Drug Administration
FP	Fundus photography
GA	Geographic atrophy
IOP	Intraocular pressure
IZ	Interdigitation zone
logMAR	Logarithm of the minimum angle of resolution
MZ	Myoid zone
*n*	Sample size
nAMD	Neovascular age-related macular degeneration
OCT	Optical coherence tomography
ONL	Outer nuclear layer
OPR	Outer photoreceptor segment
PR	Photoreceptor
RPE	Retinal pigment epithelium
SD	Standard deviation
SQRT	Square root
VA	Visual acuity
VEGF	Vascular endothelial growth factor-A
Y0	Year zero (baseline)
Y5	Year five (pegcetacoplan initiation)
